# Novel SrO-Containing Glass-Ceramic Sealants for Solid Oxide Electrolysis Cells (SOEC): Their Design and Characterization under Relevant Conditions

**DOI:** 10.3390/ma15175805

**Published:** 2022-08-23

**Authors:** Hassan Javed, Elisa Zanchi, Fabiana D’Isanto, Chiara Bert, Domenico Ferrero, Massimo Santarelli, Federico Smeacetto

**Affiliations:** 1 Sunfire GmbH, Gasanstaltstraße 2, 01237 Dresden, Germany; 2 Department of Applied Science and Technology (DISAT), Politecnico di Torino, 10129 Turin, Italy; 3 Department of Energy (DENERG), Politecnico di Torino, 10129 Turin, Italy

**Keywords:** SOEC, sealants, glass-ceramic, sintering

## Abstract

This study presents results on the development of strontium oxide (SrO) containing glass sealants used to join Crofer22APU to yttria-stabilized zirconia (3YSZ), in which the main glass components, that is, silicon oxide (SiO_2_), strontium oxide (SrO), calcium oxide (CaO) and aluminum oxide (Al_2_O_3_), have been varied appropriately. Certain properties, such as the crystallization behavior, the coefficient of thermal expansion, adhesion, and reactivity of the sealants in contact with Crofer22APU, have been reviewed and discussed. The optimized glass composition (with CTE in the 9.8–10.3 × 10^−6^ K^−1^ range) results in a good joining behavior by hindering the formation of undesirable strontium chromate (SrCrO_4_) on contact with the Crofer22APU steel after 1000 h. at 850 °C. High specific resistivity values of about 10^6^ Ohm.cm have been obtained, thus demonstrating good insulating properties at 850 °C under an applied voltage of 1.6 V. A negligible degradation in the electrical resistivity trend was measured during the test up to 1000 h, thus excluding the presence of detrimental reactions of the glass-ceramic sealant in contact with Crofer22APU under a dual atmosphere, as confirmed using SEM-EDS post-mortem analyses.

## 1. Introduction

A solid oxide cell (SOC) is a device that can work either in solid oxide fuel cell (SOFC) mode, in solid oxide electrolysis (SOEC) mode, or as a reverse solid oxide cell (rSOC) where both the SOFC and SOEC modes can work alternatively [1]. A SOFC converts chemical energy into electrical energy [2,3,4], while a SOEC uses electrical energy from different renewable sources and produces chemical energy using the direct electrolysis of water. Pure hydrogen can be produced through the direct electrolysis of water in the SOEC technology and subsequently used as an energy vector over a wide range of applications, from mobility to industry (e.g., refinery, steel manufacturing, etc.). In addition, in SOEC, a mixture of water and CO_2_ can also be co-electrolyzed to produce syngas (a mixture of hydrogen and carbon monoxide), which in turn can be utilized to synthesize a variety of hydrocarbons [5,6,7,8,9].

SOEC stacks usually operate at high working temperatures, i.e., from 700 °C to 850 °C [10,11,12,13,14]. The degradation of stack components at such working temperatures can limit the performance of SOEC components in the long term and during dynamic operation. In this context, the durability and performance of the sealants used in SOECs are one of the issues of most concern. Sealants are mainly used in SOEC stacks to avoid the mixing of gases at both electrodes, and they also provide electrical insulation to avoid short circuits in a stack. Therefore, they should be highly dense to ensure the tightness of the gases and should have a high electrical resistivity (>10^5^ Ω.cm) [15]. In addition, the sealants should have a coefficient of thermal expansion (CTE) in the 9–12 × 10^−6^ K^−1^ range to ensure a strong bond with the other cell components, such as a metallic interconnect (CTE: 10.5–12 × 10^−6^ K^−1^ [14]) and a yttria-stabilized zirconia (YSZ) electrolyte (CTE: 10.5 × 10^−6^ K^−1^), as a CTE mismatch can lead to the formation of cracks or delamination during operation. Moreover, to avoid any undesirable chemical interaction between the sealants and other cell components, the sealants should be chemically stable at high working temperatures and under humid conditions [16,17].

Glass-ceramics-based sealants are considered the most promising solution, due to their remarkable properties in terms of high thermal and chemical stability, high electrical resistivity, and their ability to form a rigid hermetic sealant. So far, most of the studies in this field have been carried out on the synthesis of suitable sealants for use in SOFC technology [3,18,19,20,21,22,23,24,25,26]. Although most of the requirements of a sealant to operate in SOECs are similar to those of SOFCs, however, the requirement of a higher electrical resistivity under the applied voltages (>1.2 V) in SOECsand the thermal cyclic conditions, especially in the case of rSOC, further narrow their selection. The properties of glass-ceramics, such as the glass transition temperature (Tg), CTE, etc., mainly depend on their compositions and can be tuned. However, due to the high working temperature and presence of harsh humid conditions, the synthesis of reliable glass-ceramic-based sealants that would allow them to operate for 30,000–40,000 h remains somewhat challenging. 

Silica (SiO_2_)-based glass-ceramics are generally used as sealants for high-temperature SOECs, where silica acts as glass former. Besides being a glass former, the choice and concentration of modifiers play an important role in controlling the overall properties of glass-ceramics. Critical modifiers such as alkali oxides should be avoided as their presence can have a negative impact on the long-term stack stability due to their high reactivity with metallic interconnects and reduction in electrical resistivity under voltage [27,28,29]. 

Although a lot of glass-ceramic compositions have been reported in the literature, however, barium oxide (BaO)-Silica (SiO_2_)-based glass-ceramic sealants have been studied the most, and they have shown promising behavior [11,18,19,26,30,31,32,33,34,35]. However, in alumino-silicate-based glass systems, BaO forms a low-CTE BaAl_2_Si_2_O_8_ celsian phase, which could lead to the generation of stresses within the glass-ceramic or at the Crofer22APU/glass-ceramic interface [19,36,37]. Moreover, BaO reacts spontaneously with Cr from high Cr steel interconnects, such as Crofer22APU, and forms a high CTE barium chromate (BaCrO_4_) phase [32,34]. Strontium oxide (SrO) is considered the most promising alternative to BaO because SrO can improve the CTE of glass, reduce viscosity and improve wettability. Many SrO-based glass-ceramic compositions are mentioned in the literature. However, there is still a lack of studies conducted to investigate and understand their long-term performance, especially in a dual atmosphere [14,38,39,40,41,42,43,44,45]. Moreover, most of the considered glass compositions were studied up to a working temperature of 800 °C. For instance, López et al. [41] investigated the mechanical properties of two different glass-ceramic systems containing BaO and SrO as modifiers before and after aging at 800 °C up to 800 h. The glass-ceramic with SrO showed better mechanical properties than the BaO-containing composition. Wang et al. [40] investigated the thermal properties of SrO–La_2_O_3_–Al_2_O_3_–SiO_2_-based glass-ceramic systems in air and steam. The glass-ceramics showed good CTE after joining and aging for 1000 h at 800 °C, but they only studied the behavior of glass-ceramics in steam up to 24 h. Chou et al. [46] examined the electrical properties of an SrO-based glass (YS046) for 500–1000 h at 800–850 °C under a DC load of 0.7 V and in a dual atmosphere (air and steam). The YS046 glass formed unwanted Sr-chromates at the glass/Crofer22APU interface after 500 h of operation. These compositions were designed to obtain the desired high-CTE SrSiO_3_ phase—that is, with a CTE of 10.9 × 10^−6^ K^−1^ [47]. According to the SiO_2_-SrO phase diagram [48], SiO_2_/SrO (mol%) should be equal to one to obtain a single SrSiO_3_ phase. However, additional factors should be considered to optimize the ratio between the glass former and glass modifier: the glass transition temperature should be able to ensure sealing processability during stack consolidation, as well as maintain self-healing properties during stack operation. Moreover, a high SrO content increases the possibility of an undesirable high-CTE SrCrO_4_ phase forming due to a chemical reaction between the SrO from the glass-ceramic and the Cr from the stainless-steel interconnect [24]. Therefore, for the long-term stability of glass-ceramic sealants, it is important to avoid the formation of high-CTE chromates. On the other hand, a high SiO_2_ content can lead to the formation of a cristobalite phase (SiO_2_) that shows volume expansion around 270 °C and the possible formation of cracks: for this reason, the balancing of additional glass network modifiers, intermediates and additives is required in the design of novel sealant compositions [45,49].

In this research work, novel silica-based glass-ceramic sealants have been designed using SrO as the main modifier for a working temperature of 850 °C: these sealants have been labeled HJ14, NS4, and NS9I. The thermo-mechanical compatibility of these glass-ceramic sealants with Crofer22APU interconnects and 3YSZ has been investigated. The electrical resistivity of the Crofer22APU/glass-ceramic/Crofer22APU joined sample was measured in a dual atmosphere for 1000 h at 850 °C under an applied voltage of 1.6 V, and this was followed by SEM-EDS post-mortem analyses.

## 2. Materials and Methods

Three novel silica-based glass-ceramic compositions were designed using SrO as the main modifier, labeled as HJ14, NS4, and NS9I. The details of the investigated compositions are shown in Table 1. 

In these glass systems, silicon oxide (SiO_2_) and boron oxides (B_2_O_3_) were added as glass network former, calcium oxide (CaO), magnesium oxide (MgO), and strontium oxide (SrO) as network modifier, aluminum oxide (Al_2_O_3_) as an intermediator, while yttrium oxide (Y_2_O_3_) as additive. The compositional range of the studied glass systems is: SiO_2_ 46–55 mol%, SrO 30–40 mol%, CaO 2–9 mol%, Al_2_O_3_ 2–7 mol%, B_2_O_3_ 3–8 mol%, Y_2_O_3_ 0–5 mol%, MgO 0–1 mol%. Table 1 reports the relevant ratios between various glass formers, network modifiers, and intermediates for the HJ14, NS4, and NS9I glass compositions. As shown in Table 1, the ratio between the glass formers and glass modifiers was set at around 1.3- to 1.5 for the three glass compositions; different ratios were obtained between the main glass former and main glass modifier by varying the SiO_2_ and SrO amounts, respectively. The SiO_2_/SrO was kept at 1.5 for the HJ14 composition; a significant amount of CaO was added to avoid the formation of the cristobalite phase, despite the high SiO_2_ content. Moreover, Al_2_O_3_ was added to hinder devitrification and adjust the viscosity of the residual glassy phase. The total concentrations of glass formers (CaO + MgO + SrO) in HJ14, NS4, and NS9I systems were kept at 44 mol%, 38 mol%, and 40 mol%, respectively. The SiO_2_ and SrO contents were adjusted to obtain ratios of 1.4 and 1.3 for NS4 and NS9I, respectively. Consequently, progressively higher ratios of B_2_O_3_/Al_2_O_3_ were chosen by reducing the alumina and increasing the former glass amount to both avoid the risk of the formation of the low-CTE celsian SrAl_2_Si_2_O_8_ phase and to increase the quantity of the residual glassy phase, despite the addition of a smaller amount of SiO_2_. The high SrO/CaO ratio in the NS4 and NS9I compositions indicates the addition of small amounts of CaO, which was replaced by the introduction of Y_2_O_3_ to adjust the glass viscosity and increase the CTE of the glass phase.

The raw materials used for glass synthesis were SiO_2_ (>99%), SrCO_3_ (>99%), CaCO_3_ (>99%), MgCO_3_ (>99%), Al_2_O_3_ (99.9%) and H_3_BO_3_ (99.99%). The glass was synthesized using the melt quenching technique, whereby the raw materials, in the form of oxides and carbonates, were homogenously mixed for one day. The mixture was then melted for one hour at 1600 °C in a Pt-Rh crucible, followed by quenching on a brass plate. The glass was then ball milled and sieved to obtain particles with a size below 25 µm.

The glass transition (Tg) and crystallization temperatures (Tp) of the glass powder were analyzed by conducting differential thermal analyses (DTA Netzsch, Eos, Selb, Germany) up to 1200 °C at a heating rate of 5 °C/min. The sintering behavior of glass was analyzed using a heating stage microscope (HSM Expert system solutions, Modena, Italy) at temperatures of up to 1200 °C and at a heating rate of 5 °C/min to observe the first sintering temperature (TFS) and maximum sintering temperature (TMS). The coefficient of thermal expansion (CTE) of the glass and the glass-ceramic was investigated using a dilatometer (Netzsch, DIL 402 PC/4) at temperatures of up to 1000 °C and a heating rate of 5 °C/min. The dilatometer was used to perform measurements on the as-cast bulk glass after polishing with SiC paper to obtain two opposite plane parallel sides with a thickness of 5 mm; a cylindrical pellet (10 mm in diameter) was prepared for the glass-ceramic by sintering pressed glass powder and then polishing it to obtain a final thickness of 5 mm. The CTE of the glass-ceramic was also measured after aging for 1000 h at 850 °C in static air. Three measurements were performed for each characterization (DTA, HSM, and Dilatometer) to ensure reproducibility and to obtain statistical data. 

The different crystalline phases of the glass-ceramic were analyzed using XRD-PANalytical X’Pert Pro PW 3040/60 Philips (The Netherlands), with CuKα and the X’Pert software. The XRD analyses were carried out in the 2 theta 10°–70° range, with a step size of 0.02626° and a time per step of 10.20 sec. XRD was performed on the as-sintered and thermally aged (1000 h, 850 °C) glass-ceramic sintered pellets to analyze the formation of different crystal phases.

To investigate the compatibility of the glass-ceramic with Crofer22APU and 3YSZ, a Crofer22APU/glass-ceramic/3YSZ joined sample was produced in a furnace (CWF 13/5, Carbolite) in static air. Before joining, the Crofer22APU and 3YSZ substrates, which had dimensions of 1.5 cm × 1.5 cm, were cleaned with acetone. The glass was deposited manually in the form of a slurry composed of glass powder and ethanol. The cross-section of the Crofer22APU/glass-ceramic/3YSZ interface was investigated using a scanning electron microscope (FESEM, Merlin ZEISS, Munich, Germany). For that purpose, the cross-section was polished, up to 1 µm, using diamond paste and coated with gold for SEM investigation. EDS point analyses were carried out to observe the composition of different phases in the glass-ceramic, in addition to the EDS line scan, to observe any possible diffusion of elements across the Crofer22APU/glass-ceramic interface. 

The evaluation of the preliminary investigations allowed us to identify HJ14 as the most promising material for subsequent electrical characterization in the SOEC atmosphere. 

The electrical resistivity of the Crofer22APU/HJ14 glass-ceramic/Crofer22APU was measured in-situ at 850 °C under a dual atmosphere and applied voltage. The glass was deposited, in the form of slurry, on a cleaned Crofer22APU plate (3 cm × 6 cm × 0.2 cm) to form a closed sealing frame and joined to a second Crofer22APU plate of the same size. The lower plate had two holes to allow the inlet and outlet of a controlled atmosphere during the experiment, while the external side of the glass sealing was exposed to static air. A mixture of 50 mol% hydrogen and 50 mol% steam was sent to the joint sample during the experiment. A uniformly distributed weight was placed on the top plate. The joining treatment described in Section 3.1 was applied before settling the temperature to 850 °C and exposing the sample to the dual atmosphere. A voltage of 1.6 V was applied between the upper and lower plates, which were connected to a voltage generator and a measuring circuit by platinum wires. Further details about the testing methodology and resistivity measurements can be found elsewhere [50]. 

After the resistivity test, SEM-EDS post-mortem analyses were carried out to investigate the Crofer22APU/HJ14 glass-ceramic/Crofer22APU joint in contact with air and humid conditions.

## 3. Results and Discussion

### 3.1. Thermal Analysis

**Figure 1:**
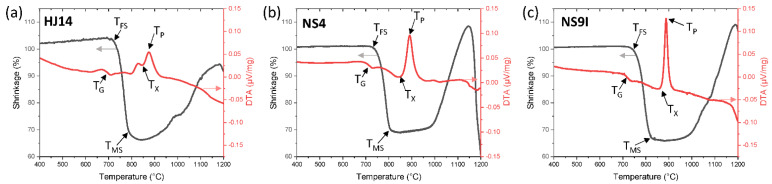
DTA vs. HSM curves for the HJ14 (**a**), NS4 (**b**) and NS9I (**c**) glass systems, from analyses carried out at a heating rate of 5 °C/min.

Figure 1 shows the results of the DTA and HSM analyses carried out on the HJ14 (a), NS4 (b), and NS9I (c) glass powders (<25 µm) at temperatures of up to 1200 °C at a heating rate of 5 °C/min. In Figure 1, the Tg, Tx, and Tp labels on the DTA curve correspond to the glass transition temperature, the onset of the crystallization temperature, and the peak crystallization temperature, respectively. The T_FS_ and T_MS_ labels on the HSM curve (Figure 1) represent the first sintering and maximum sintering temperatures, respectively. The corresponding characteristic temperatures are also summarized in Table 2.

As reported in Table 2, the HJ14 glass showed a Tg of 695°and a Tp of 876 °C, respectively, as measured utilizing DTA. The sintering process was initiated at 716 °C, as measured using HSM. The shrinkage continued beyond the first sintering temperature (T_FS_) until it attained a maximum shrinkage at 820 °C. Both the NS4 and NS9I glasses expressed a comparable Tg, but a significantly higher T_FS_, thus demonstrating the more viscous behavior of these two compositions than HJ14. On the other hand, the T_MS_ of studied glass systems also differed slightly, but all three compositions reached a similar maximum shrinkage value, followed by a plateau in the HSM curve. Moreover, Tx and Tp for three glasses were comparable, thus indicating crystallization of the same phase.

To obtain good densification of a sealant, it is necessary that sintering is completed before the beginning of the crystallization process; as soon as crystallization occurs, glass viscosity increases drastically, thus hindering the viscous flow of the glass. Therefore, the crystallization mechanism of the glass-ceramic should be controlled and considered in the heat treatment schedule [51]. From the data given in Table 2, it is clear that the sintering process was completed before the start of the crystallization for all the HJ14, NS4, and NS9I glass systems. On the basis of the DTA and HSM data, a sinter-crystallization treatment between 900 and 950 °C was chosen as the optimum one to obtain a dense glass-ceramic sealant for the three glass systems. The coefficients of thermal expansion (CTEs) of the as-joined glass-ceramics for the studied glass systems are shown in Table 3. The as-joined glass-ceramics showed a CTE in the range of 9.9–10.3 × 10^−6^ K^−1^, thus closely matching other cell components, and is suitable for SOEC applications. Moreover, the CTEs of these glass systems are higher than the CTEs of previously studied similar SrO-based glasses [14], thanks to the formation of suitable crystalline phases (see Section 3.2).

### 3.2. XRD Analysis of the Crystalline Phases

**Figure 2:**
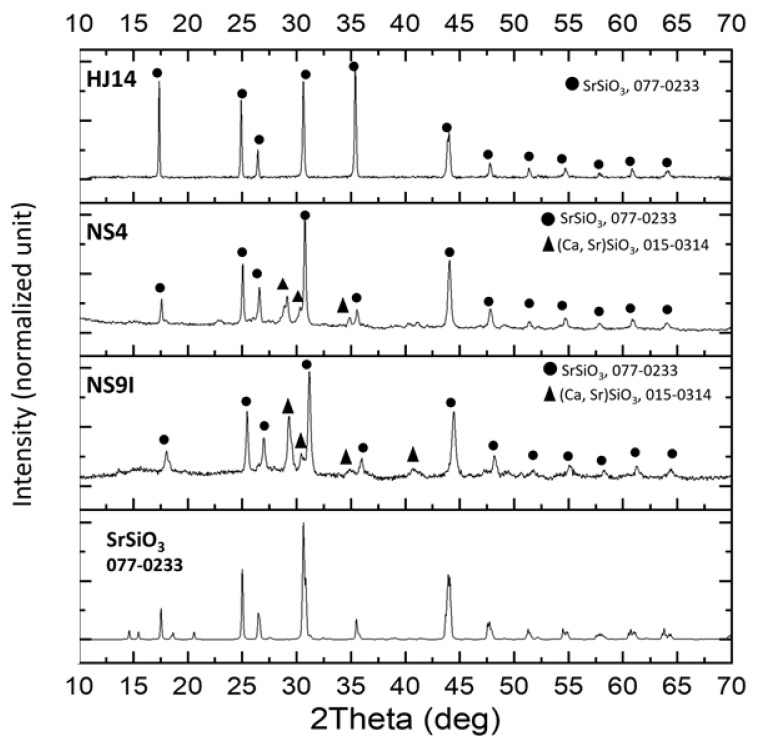
XRD patterns of the HJ14, NS4, and NS9I glass-ceramics after joining.

The results of the XRD phase analyses performed on the as-joined HJ14, NS4, and NS9I glass-ceramics are shown in Figure 2. SrSiO_3_ was detected as the only crystalline phase in the as-joined HJ14 glass-ceramics. The XRD pattern of the SrSiO_3_ phase (reference number: 01-077-0233) retrieved from the X’P database is also shown in Figure 2 for comparison purposes. The formation of only a SrSiO_3_ phase validated the rationale behind the design of the HJ14 composition, i.e., to produce a high CTE SrSiO_3_ phase and to avoid the formation of low CTE celsian (SrAl_2_Si_2_O_8_), as well as cristobalite (SiO_2_) phases. 

SrSiO_3_ is also the main crystalline phase in the NS4 and NS9I glass-ceramics. However, two additional peaks (at around 2Theta = 29.4 and 30.4) can be detected and are likely attributable to the main peaks of calcium-strontium silicate (PDF2 015-0314). 

### 3.3. Morphological and Compositional Analysis

**Figure 3:**
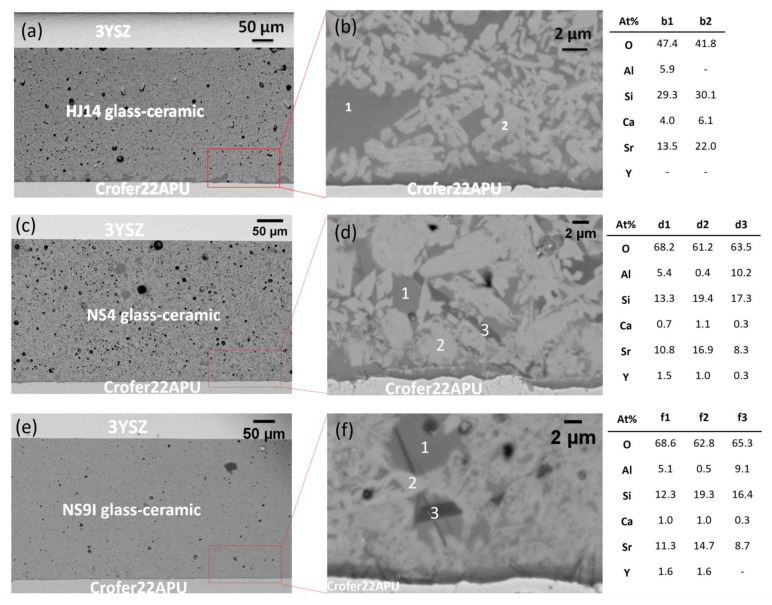
SEM backscattered electron images of the as-joined Crofer22APU/glass-ceramic/3YSZ joined samples at different magnifications, together with an EDS elemental analysis (at.%) of the marked points: HJ14 glass-ceramic (**a**,**b**); NS4 glass-ceramic (**c**,**d**); NS9I glass-ceramic (**e**,**f**).

Figure 3 shows the SEM images of the Crofer22APU/ glass-ceramic/3YSZ joined samples, processed in the 900–950 °C temperature range for 2 h. The comparison of the images at lower magnification in Figure 3, i.e., for HJ14 (Figure 3a), NS4 (Figure 3c), and NS9I (Figure 3e), points out that the selection of a suitable joining treatment has led to a very dense glass-ceramic morphology and a crack-free sealing in all three cases. The interface between the sealant and either the steel or 3YSZ demonstrates good adherence and compatibility without any delamination at either interface. However, a closer inspection of the SEM images at a higher magnification reveals different microstructures in the glass-ceramic sealants. The HJ14 glass-ceramic (Figure 3b) contains a uniform distribution of crystals and the residual glassy phase. EDS analysis of the marked points has confirmed that the dark phase (point b1) corresponds to the residual glassy phase and contains all the constituent elements of the HJ14 glass; the bright phase (point b2) shows an Sr- and Si-rich phase and thus refers to the SrSiO_3_ phase detected in the XRD analysis. However, it is apparent that some diffusion of Ca occurred in the SrSiO_3_ phase, as shown in EDS for point 2. When comparing the Sr amount at points b1 and b2, strontium is mainly located in the crystalline phase, which is beneficial for minimizing the possibility of forming an undesirable SrCrO_4_ phase in the long term. Additionally, a minimal amount of Sr in the residual glassy phase is important to maintain viscous flow behavior in the residual glassy phase.

On the other hand, the NS4 glass-ceramic (Figure 3d) shows a prominent presence of crystalline phases and a low residual glass phase. The EDS analysis has confirmed the formation of the SrSiO_3_ phase (point d2), which is recognizable as the light-gray crystals. However, the elemental composition of the glassy phase has revealed a still large amount of Sr. This observation is in line with compositional data of the starting glasses (Table 1) since the SiO_2_/SrO ratio is lower for NS4 than for HJ14. Moreover, a second crystalline phase is visible in Figure 3d as a dark-gray needle-like crystal. EDS analysis at point d3 has shown the presence of Al in addition to Sr and Si, thus indicating the possible formation of a SrAl_2_Si_2_O_8_ celsian phase. Similar considerations are valid for the NS9I glass-ceramic composition. As shown in Figure 3f, a very small quantity of the residual glass phase is visible, and the observed high degree of crystallization is in line with the findings of the DTA and dilatometric analyses. The EDS compositional analyses of points f1 (glassy phase) and f2 (crystalline phase) correspond to the results of the NS4 composition, thus confirming that SrSiO_3_ is the main phase and that there is a high SiO_2_/SrO ration in the residual glass. The elemental analysis at point f3 confirms the formation of low-CTE celsian SrAl_2_Si_2_O_8_. When compared with the NS4 composition, it was found that, in this case, the needle-like dark-gray crystals were also present at the interface with Crofer22APU. 

The development of the SrAl_2_Si_2_O_8_ celsian phase could be detrimental to the long-term operation of the sealant, especially when it develops at the interface with the steel interconnect. In the case of the three glasses presented here, it is apparent that it preferentially forms when the SiO_2_/SrO ratio in the staring compositions is progressively reduced (from HJ14 to NS4 and to NS9I), despite the parallel decrease of the relative Al_2_O_3_ content. For these reasons, HJ14 was selected as the most promising sealing material. It was then subjected to aging in a dual atmosphere to further assess and evaluate its functional properties under relevant conditions.

### 3.4. Electrical Resistivity Analyses in a Dual Atmosphere

**Figure 4:**
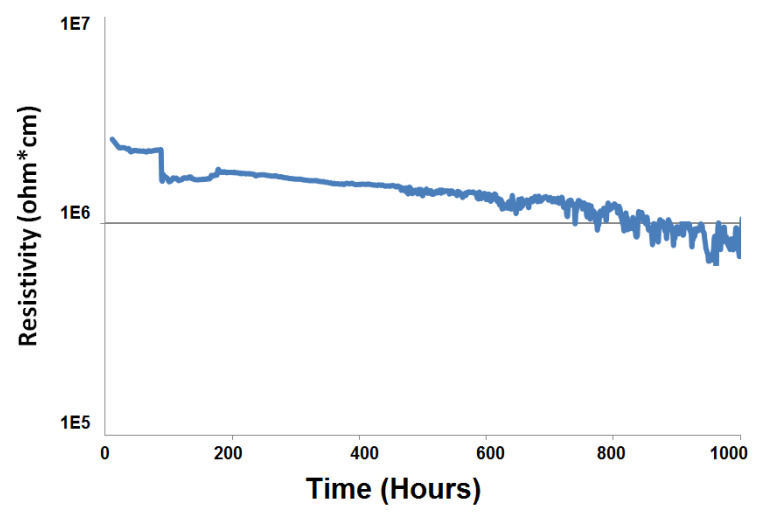
Electrical resistivity of the Crofer22APU/HJ14 glass-ceramic/Crofer22APU joined sampled, measured after 1000 h at 850 °C in a dual atmosphere.

Figure 4 shows the electrical resistivity curve of the Crofer22APU/HJ14 glass-ceramic/Crofer22APU joined sample measured under an applied voltage of 1.6 V for 1000 h at 850 °C in a dual atmosphere. The electrical resistivity values of the HJ14 glass-ceramic-based joint are higher than 1 × 10^5^ Ω.cm. They are comparable with the electrical resistivity of other glass-ceramics discussed in the literature [52] when tested under a dual atmosphere. A high electrical resistivity ensures the insulating behavior of the HJ14 glass-ceramic sealant sandwiched between the two conducting Crofer22APU plates, thus excluding the possibility of a short circuit. The interaction of the glass-ceramic with the Crofer22APU interconnect can lead to the possibility of short-circuiting and the consequent degradation of the cell. The presence of a dual atmosphere, a high working temperature, and the applied voltage not only facilitates this interaction but also cause the formation of some conductive species, such as Fe_3_O_4_ and FeO, due to the volatilization of iron from Crofer22APU [16,17]. However, in the case of the HJ14 glass-ceramic-based joint, no short circuit was observed during the electrical resistivity measurements, even in the presence of a high steam content, a high working temperature (850 °C) and an applied voltage of 1.6 V. Furthermore, no formation of iron-based (Fe_3_O_4_ and FeO) oxides or interaction of the glass-ceramic with Crofer22APU was detected during the post-mortem analyses, as discussed in Section 3.5. 

The electrical resistivity trend reported in Figure 4 shows some irregularity around approximately 100 h, most likely due to some polarization effect. Afterward, the electrical resistivity values showed almost linearly decreasing behavior. This trend is similar to that of other glass-ceramics tested under SOFC voltage conditions for shorter periods [53], with the resistivity of some of the samples reaching a plateau within 300 h of testing [53]. Although, after testing for 1000 h, the electrical resistivity of the HJ14 glass-ceramic-based joint is much higher than the threshold (10^5^ ohm.cm) for SOEC applications. However, a longer test would be needed to investigate whether the resistivity of HJ14 could also evolve toward an asymptotic value after a longer exposition to dual atmosphere conditions and applied voltage.

### 3.5. Post-Mortem Analyses

Along with the electrical resistivity test of the HJ14 glass-ceramic-based joint, the sintered HJ14 glass-ceramic pellets were thermally aged for 1000 h at 850 °C, and subsequently the CTE and XRD analyses were conducted. The HJ14 glass-ceramic showed a CTE of 9.8 ± 0.1 × 10^−6^ K^−1^ after aging, thus a slight reduction (0.5 ±0.1 × 10^−6^ K^−1^). However, this reduction was negligible, so it should not affect the performance of the glass-ceramic sealant in SOEC applications.

In general, the change in the CTE of the glass-ceramic after thermal aging is likely due to the formation of new crystalline phases. However, in the case of HJ14, no new phases were formed due to aging (1000 h, 850 °C), as confirmed by XTD analysis (see Appendix A). The XRD patterns of the HJ14 glass-ceramic after thermal aging were similar to the as-joined HJ14 glass-ceramic, with no new peak, thus confirming that the HJ14 system is stable after aging.

The SEM-EDS post-mortem analyses were carried out on the Crofer22APU/HJ14 glass-ceramic/Crofer22APU joined sample to investigate the thermo-mechanical compatibility of the glass-ceramic with Crofer22APU and the possible formation of chromates (or other undesired crystalline phases) at the Crofer22APU-glass-ceramic-air triple phase boundary. SEM-EDS analyses were performed on air sides after testing the joined sample under an electric load at 850 °C for 1000 h. 

**Figure 5:**
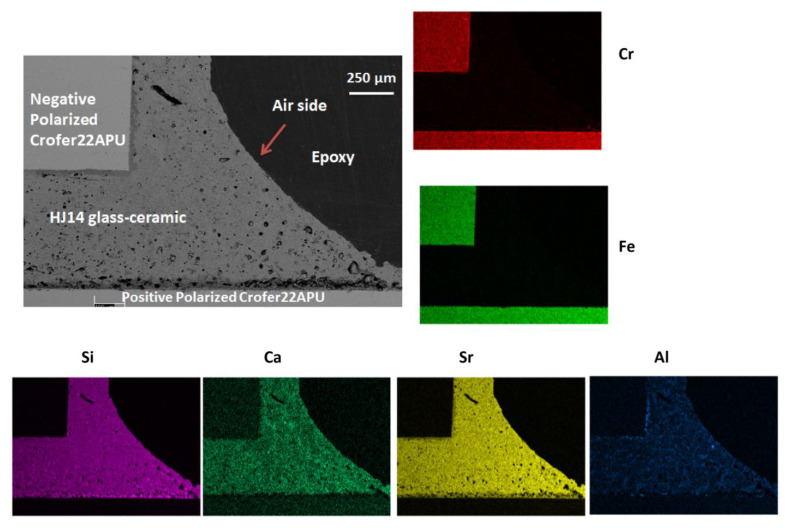
SEM post-mortem analyses of the air side of the Crofer22APU/HJ14 glass-ceramic/Crofer22APU joined samples after a thermal aging of 1000 h at 850 °C under an applied voltage of 1.6 V.

Figure 5 shows the air side of the Crofer22APU/HJ14 glass-ceramic/Crofer22APU joined sample during the electrical resistivity test. Good interfacial bonding and thermo-mechanical compatibility between the Crofer22APU and the HJ14 glass-ceramic are evident in Figure 5. The glass-ceramic is quire dense throughout the joining area.

**Figure 6:**
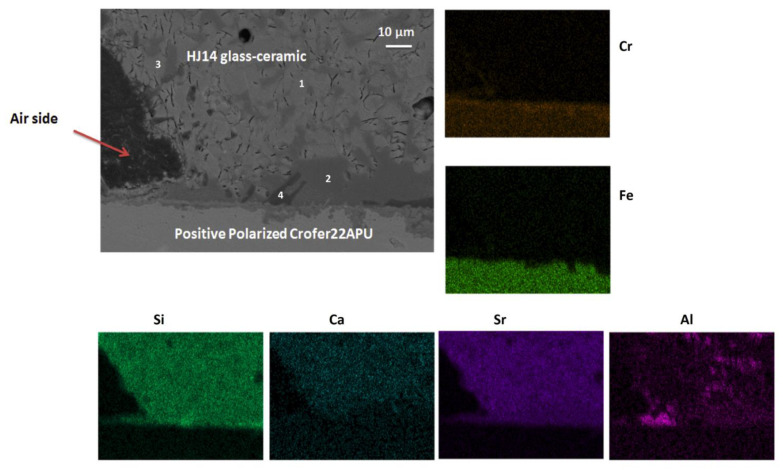
SEM image and EDS mapping of the air side of the positively polarized Crofer22APU/HJ14 glass-ceramic interface.

Figure 6 shows the magnified SEM image and EDS mapping of the air side of the positively polarized Crofer22APU/HJ14 glass-ceramic interface. No cracks within the HJ14 glass-ceramic or delamination were observed at the Crofer22APU/glass-ceramic interface. The different crystals are uniformly dispersed (bright regions) in the HJ14 glass-ceramic. The results of the corresponding EDS point analyses performed on these different phases in the HJ14 glass-ceramic are given in Table 4. The EDS analyses carried out at point 1 (Figure 6) correspond to the SrSiO_3_ phase, thus validating the XRD analyses, as discussed in Section 3.2. However, some diffusion of Ca in the SrSiO_3_ phase was also detected by the EDS, in the same way as in the EDS analyses performed on the as-joined glass-ceramic. The EDS analyses at Point 2 (Figure 6) correspond to the residual glassy phase, as it contains all the constituent components of the HJ14 glass system.

In contrast with point 1, a lower concentration of Ca was detected in the residual glassy phase due to its diffusion in the SrSiO_3_ phase. The residual glassy phase also contains 9 at.% Sr, which is beneficial for maintaining the glass transition temperature (Tg) and for producing viscous behavior in the residual glass. A negligible concentration of Cr (0.3 atm %) was also detected in the residual glassy phase, close to the Crofer22APU interface. The residual glassy phase (dark regions) is mainly present along the Crofer22APU substrate and is beneficial for promoting self-healing at the Crofer22APU/ glass-ceramic interface above Tg. The EDS analysis at point 3 is similar to that at point 1, thus indicating the presence of SrSiO_3_, with a slight diffusion of Ca. A black phase was also observed by means of SEM at the Crofer22APU/glass-ceramic interface, as indicated by point 4. The corresponding EDS at point 4 (Table 4) confirms that this phase corresponds to a cristobalite phase (SiO_2_). However, the SEM image of HJ14 (Figure 6) shows that the concentration of cristobalite is negligible and is only crystallized along the Crofer22APU substrate.

The EDS mapping shown in Figure 6 and the EDS point analyses performed at different regions on the HJ14 glass-ceramic joined to the positively polarized Crofer22APU did not detect the diffusion of Cr, excluding the possibility of the formation of an undesirable SrCrO_4_ phase.

**Figure 7:**
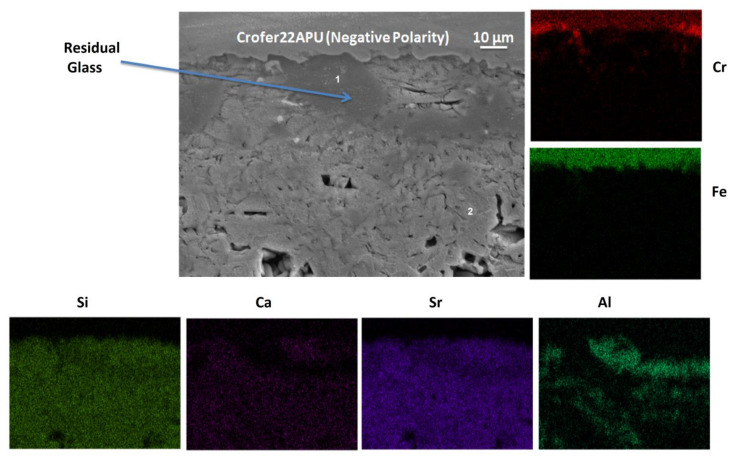
SEM image and EDS mapping of the air side of the negatively polarized Crofer22APU/HJ14 glass-ceramic interface.

The SEM image and EDS mapping of the negatively polarized Crofer22APU/HJ14 glass-ceramic interface on the air side are shown in Figure 7. A strong bonding was observed between the negatively polarized Crofer22APU and the HJ14 glass-ceramic, with no delamination. The glass-ceramic seems significantly dense (with little porosity), ensuring gas tightness during SOEC operation. The results of the corresponding EDS analyses performed in different regions are given in Table 5. The EDS analyses at point 1 in Figure 7 correspond to the residual glassy phase, mainly present close to the interface with Crofer22APU. The concentrations of Sr, Si, and Ca in the residual glassy phase are comparable on both sides of the polarized Crofer22APU (Table 5). However, a slight Cr content (1.2 at.%) was observed in the residual glassy phase at the negative polarized Crofer22APU/HJ14 glass-ceramic interface. Nevertheless, like the positive polarized Crofer22APU/HJ14 glass-ceramic interface, no chromates were detected at the negative polarized Crofer22APU/HJ14 glass-ceramic interface, thus excluding the possibility of any corrosion. The EDS analysis at point 2 shows the presence of an SrSiO_3_ phase, with a slight diffusion of Ca.

**Figure 8:**
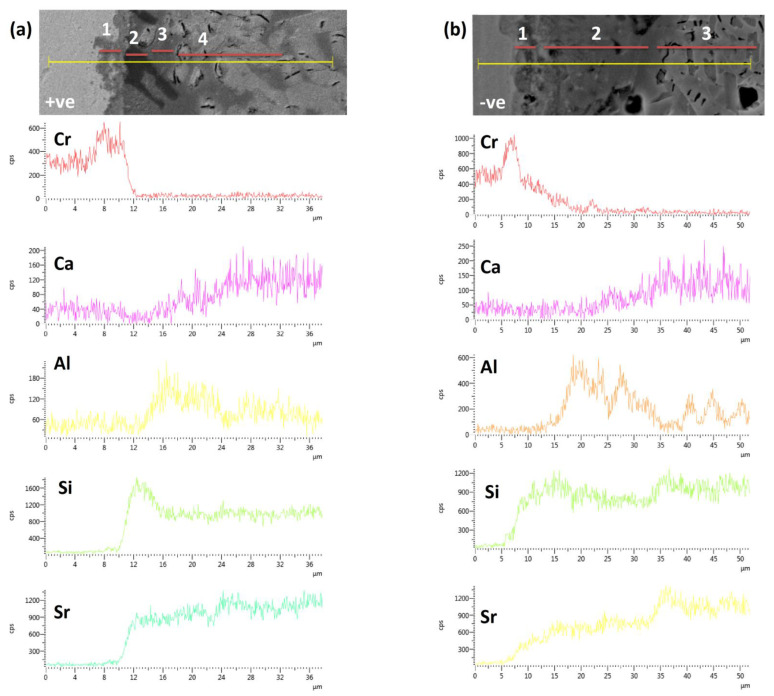
EDS line scan across (**a**) the air side of the positive polarized Crofer22APU/HJ14 glass-ceramic and (**b**) the air side of the negative polarized Crofer22APU/HJ14 glass-ceramic interfaces.

Figure 8 compares the EDS line scan performed across the Crofer22APU/HJ14 glass-ceramic interface, where the Crofer22APU has positive (Figure 8a) and negative polarities (Figure 8b). The interfaces shown in Figure 8 both correspond to the air side. A direct comparison shows that, in the case of the negatively polarized Crofer22APU, there has been a diffusion of Cr from the Crofer22APU into the glass ceramic. In contrast, no Cr diffusion can be detected from the positively polarized Crofer22APU. On the other hand, no diffusion or segregation of the glass-ceramic elements can be detected across the Crofer22APU/HJ14 glass-ceramic interface. These results are in agreement with the results of the EDS point analyses given in Table 4 and Table 5, where a slight amount of Cr diffusion was detected from the negative polarized Crofer22APU on the glass-ceramic side.

Figure 8 shows that the Crofer22APU/HJ14 glass-ceramic interface can be divided into different regions based on the different phases. In Figure 8a, region 1 corresponds to a Cr-oxide scale of ~3 µm thickness. The formation of the oxide scale is as expected and is formed during high-temperature aging. Region 2 (black phase) shows a high concentration of Si and corresponds to the cristobalite phase, as discussed in Section 3.5. Region 3 is the residual glassy phase, as it contains a high concentration of Al, Si, and Sr, in addition to a small concentration of Ca. The concentration of Al is reduced in region 4, compared to region 3, although it contains a significantly high concentration of Si and Sr, and thus refers to the SrSiO_3_ phase. Moreover, the concentration of Ca also increases in region 4 compared to region 3, thus confirming its diffusion in the SrSiO_3_ phase, as discussed for the EDS analyses in Section 3.3. 

Similarly, for Figure 8b, region 1 is a Cr-rich area and thus refers to the Cr-oxide scale. Region 2 has a high concentration of Al, Si, and Sr, in addition to a small quantity of Ca, corresponding to the residual glassy phase. The Si and Sr concentration in region 3 has increased, while the Al concentration has reduced compared to region 2; thus, region 3 mainly contains the SrSiO_3_ phase. As in Figure 8a, the concentration of Ca is higher in the SrSiO_3_ region than in the residual glass due to its possible diffusion during thermal aging.

## 4. Conclusions

The newly developed SrO-based glass-ceramic sealants were characterized for solid oxide electrolysis cell (SOEC) applications at a working temperature of 850 °C. This study showed that in SiO_2_-SrO-Al_2_O_3_-based glass-ceramic systems, the relative ratios between SiO_2_, SrO, and Al_2_O_3_ must be chosen carefully to form a high CTE SrSiO_3_ phase and to avoid the formation of undesirables phases, i.e., SrCrO_4_, cristobalite (SiO_2_) and celisan. Of the three studied glass-ceramics, the HJ14 system showed the most promising results in terms of high density, suitable CTE, and absence of undesirable phases, which ensured excellent thermo-mechanical compatibility of the HJ14 glass-ceramic with the Crofer22APU interconnect and 3YSZ. A high electrical resistivity (>10^5^ Ω.cm) was measured for the HJ14 glass-ceramic sandwiched between the two conductive Crofer22APU plates, thus eliminating the possibility of a short circuit. No formation of undesirable chromates or diffusion of elements across the Crofer22APU/glass-ceramic interface was detected after post-mortem analyses of the Crofer22APU/glass-ceramic/Crofer22APU joined samples treated in a dual atmosphere for 1000 h at 850 °C. As a result of these properties, the HJ14 glass system can be considered a promising candidate for the SOEC sealant at 850 °C. However, to understand the long-term degradation behavior of HJ14 glass-ceramic, it is important to test it in the real SOEC and/or co-SOEC stacks for the long term.

## Figures and Tables

**Table 1:** Relevant correlations between the different components of the glass systems.

Glass ID	$\frac{SiO_{2}+B_{2}O_{3}}{SrO+CaO+MgO}$	$\frac{SiO_{2}}{SrO}$	$\frac{B_{2}O_{3}}{Al_{2}O_{2}}$	$\frac{SrO}{CaO+MgO}$
HJ14	1.3	1.5	3	4
NS4	1.5	1.4	2	12
NS9I	1.4	1.3	3	12

**Table 2:** Characterization temperatures of the HJ14 glass as measured by DTA and HSM (measurements carried out at 5 °C/min).

Glass ID	Glass Transition Temperature T_g_ (°C)	First Shrinkage Temperature T_FS_ (°C)	Maximum Shrinkage Temperature T_MS_ (°C)	Onset Crystallization Temperature T_x_ (°C)	Peak Crystallization Temperature T_p_ (°C)
HJ14	695 ± 3	716 ± 2	820 ± 3	854 ± 5	876 ± 5
NS4	702 ± 2	743 ±2	830 ± 3	858 ±3	890 ± 2
NS9I	707 ± 5	746 ± 2	832 ± 5	850 ± 5	887 ± 3

**Table 3:** Coefficient of thermal expansion (CTE) of the HJ14, NS4, and NS9I glass-ceramic in an as-joined condition.

Glass ID	CTEs of as-Joined Glass-Ceramic
HJ14	(10.3 ± 0.2) × 10^−6^ K^−1^
NS4	(9.9 ± 0.2) × 10^−6^ K^−1^
NS9I	(10.3 ± 0.1) × 10^−6^ K^−1^

**Table 4:** EDS point analyses (at.%) performed on HJ14 glass-ceramic bonded to positively polarized Crofer22APU on the air side (analysis corresponds to Figure 6).

	O	Al	Si	Ca	Cr	Sr
Point 1	57.2	---	21.9	4.3	---	16.5
Point 2	66.9	3.1	20.1	0.7	0.3	8.9
Point 3	54.6	---	24.0	4.4	---	17.0
Point 4	66.5	---	33.5	---	---	---

**Table 5:** EDS point analyses (at.%) performed on the air side of the HJ14 glass-ceramic bonded to the positively polarized Crofer22APU (analysis corresponds to Figure 7).

	O	Al	Si	Ca	Cr	Sr
Point 1	58.5	11.8	20.1	0.7	1.2	7.4
Point 2	56.8	0.0	23.0	4.6	0.0	15.7

**Figure A1:**
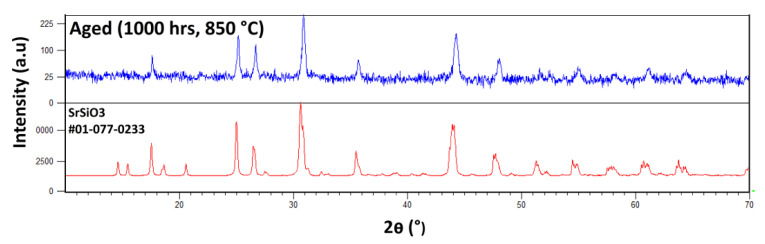
XRD analysis of thermally aged (1000 h, 850 °C) HJ14 glass-ceramic.

## Data Availability

Not applicable.

## Appendix A

XRD analysis of HJ14 after thermal aging for 1000 h at 850 °C is shown below. The aged HJ14 glass-ceramic showed the presence of only the SrSiO_3_ phase, similar to the as-joined HJ14 glass-ceramic.
